# Eicosapentaenoic acid and 5-HEPE enhance macrophage-mediated Treg induction in mice

**DOI:** 10.1038/s41598-017-04474-2

**Published:** 2017-07-04

**Authors:** Toshiharu Onodera, Atsunori Fukuhara, Jihoon Shin, Tomonori Hayakawa, Michio Otsuki, Iichiro Shimomura

**Affiliations:** 1 Department of Metabolic Medicine, Osaka University Graduate School of Medicine, 2-2, Yamamdaoka, Suita, Osaka, Japan; 2 Department of Diabetes Care Medicine, Osaka University Graduate School of Medicine, 2-2, Yamadaoka, Suita, Osaka, Japan; 3 Osaka University Graduate School of Frontier Biosciences, 2-2, Yamamdaoka, Suita, Osaka, Japan

## Abstract

Eicosapentaenoic acid (EPA) is an omega-3 fatty acid with immunomodulatory and anti-inflammatory effects. Beyond its direct effects, the metabolic products of EPA also regulate various immune responses. Animal experiments demonstrated that EPA reduces adipose inflammation in high fat diet-induced obese mouse. However, the effects of EPA on infiltrated immune cell populations in adipose tissue and underlying mechanisms remain to be elucidated. We performed flow cytometry of stromal vascular fraction of epididymal adipose tissues from C57BL/6J and ob/ob mice fed normal chow mixed with or without 5% EPA. The numbers of hematopoietic cells, including Tregs, were higher in both C57BL/6J and ob/ob mice fed EPA diet compared with control diet. EPA enhanced the induction of Tregs in co-cultures of adipose tissue macrophages (ATMs) and naïve T cells. Among EPA metabolites, 5-HEPE was the most potent inducer of Tregs. GPR119 and GPR120 are receptors for 5-HEPE and EPA, respectively, and antagonist of GPR119 blocked Treg induction by EPA in the presence of ATMs. Alox5 gene encodes 5-lipoxygenase enzyme catalyzing EPA into 5-HEPE, and inhibitor of 5-lipoxygenase down-regulated EPA-mediated induction of adipose tissue Tregs in ob/ob mice. The study findings demonstrated that both EPA and 5-HEPE enhance ATM-mediated Treg induction.

## Introduction

Accumulation of visceral adipose tissue is the main pathology of obesity, which is associated with chronic inflammation of adipose tissues^1^. The pathological process of chronic inflammation of obese adipose tissue involves several factors, such as dysregulation of adipocytokines^2^, endoplasmic reticulum (ER) stress^3^, accelerated infiltration of inflammatory macrophages, CD8^+^ T cells, and low proportion of anti-inflammatory immune cells such as regulatory T cells (Tregs) and eosinophils^4, 5^. Tregs play a pivotal role in immunological tolerance and regulate excess immune responses associated with allergy^6^, infection^7^ and tumor immunity^8^. Tregs are markedly reduced in epididymal fat of obese animals, and the reduction is closely associated with insulin resistance^4^. We recently reported the importance of adipose tissue macrophages (ATMs) in Treg differentiation and proliferation^9^.

Chronic inflammation of the adipose tissue depends on the intake of certain dietary fatty acids. For example, saturated fatty acids and trans fatty acid cause chronic inflammation in adipose tissues^10–12^, while omega-3 polyunsaturated fatty acids, such as docosahexaenoic acid (DHA) and eicosapentaenoic acid (EPA), attenuate inflammatory response, including rheumatoid arthritis^13^, asthma^14^ and inflammatory bowel disease^15^. At cellular level, EPA blocks dendritic cell activation^16^ and is associated with increased level of Tregs in EPA-treated cardiac allograft recipients^17^. In adipose tissues, EPA lowers the expression of inflammatory cytokines, such as monocyte chemoattractant protein-1 (MCP-1)^18^. EPA prevents and reverses insulin resistance in high-fat diet-induced obese mice^19^. The anti-inflammatory effects of EPA are mediated through reduction of arachidonic acid- derived inflammatory mediators, activation of nuclear receptor peroxisome proliferator-activated receptor γ (PPARγ) and G protein coupled receptor (GPR) 120^20^ as an agonist, and stimulation of the AMPK/SIRT pathway^21^. Beyond the direct effects of EPA, its metabolic products, resolvin E1 and 12-HEPE, prevent chemotaxis of antigen presenting cells, such as dendritic cells and macrophages^22, 23^. 5-HEPE, another metabolic product of EPA, stimulates insulin secretion from pancreatic β cells^24^ and glucagon like peptide-1 secretion from intestinal L cells^25^ via GPR119. With regard to the effect of EPA on infiltrated immune cell populations in adipose tissue, it is known to increase ATMs in leptin-deficient ob/ob mice and attenuates lipopolysaccharides (LPS)-dependent inflammatory response in RAW264.7 macrophages^26^. However, the effect of EPA on adipose tissue Tregs as well as other immune cells remains to be elucidated.

The present study was designed to determine the underlying mechanisms of various effects of EPA on infiltrated immune cell populations in the adipose tissue. For this purpose, we performed flow cytometry analysis of the stromal vascular fraction (SVF) of epididymal adipose tissues from EPA-treated mice, and *in vitro* analysis of adipose tissue macrophages and naïve T cells co-cultured in the presence of EPA or its metabolites. The results showed that both EPA and 5-HEPE enhance ATM-mediated Treg induction.

## Research Design and Methods

All methods were performed in accordance with relevant guidelines and regulations of Osaka University.

### Materials

Highly purified EPA (purity: 98%) ethyl ester was provided by Mochida Pharmaceutical Co., Ltd., which is used in animal and clinical studies^27^. EPA metabolites (5-HEPE, 12-HEPE, 15-HEPE, 17(18)-EpETE and prostaglandin D3) were purchased from Cayman chemical. Inhibitors for EPA metabolism (Zileuton, Etodolac and Baicalein) and PSN375963 and TUG-891 were purchased from Sigma-Aldrich. GPR119 antagonist TM43718 was purchased as E897-0145 from ChemDiv (San Diego, CA, USA).

### Animals

Male C57BL6/J and ob/ob mice were purchased from Charles River Japan (Yokohama, Japan) and used at 11–16 weeks of age. Foxp3 bicistronic reporter knock-in mice expressing EGFP were kindly provided by Dr. Kiyoshi Takeda (Osaka University, Japan). All mice were maintained under specific pathogen-free conditions and housed in groups of three per cage, maintained in a room under controlled temperature (23 ± 1.5 °C) and humidity (45 ± 15%) on a 12-h dark/12-h light cycle, and had free access to water and chow (MF; Oriental Yeast, Tokyo, Japan). The study protocol was approved and carried out in accordance with the Institutional Animal Care and Use Committee Guidelines of Osaka University.

### Administration of EPA in normal chow fed mice and ob/ob mice

5-week old C57BL6/J mice, ob/ob mice were fed for 5 weeks, *ad libitum*, AIN93g (fish oil-free diet) purchased from Charles River Japan (Yokohama, Japan) mixed with or without 5% EPA. ALOX5 inhibitor CJ13610 (Sigma) was added to the food at a dose of 5 mg/kg. Unconsumed diet was replaced with fresh one every two days. After 4–5 weeks, animals were sacrificed and adipose tissue was harvested and minced for immune cell isolation or frozen in N_2_. EPA was a generous gift from Mochida Pharmaceuticals Co., Ltd.

### Quantitative real-time PCR

Total RNA was prepared from freshly isolated cells and tissues using RNAprotect Cell Reagent (Qiagen, Hilden, Germany) according to the protocol supplied by the manufacturer. The RNA was purified using RNeasy microkit (Qiagen) based on the instructions provided by the manufacturer. The cDNA was synthesized using the Transcriptor First Strand cDNA Synthesis Kit (Roche, Indianapolis, IL). Real-time PCR was performed on the LightCycler system (FastStart DNA Master SYBR Green I, Roche) according to the protocol provided by the manufacturer. Supplementary Table 1 lists the sequences of primers used in this study.

### Treg induction in the presence or absence of adipose tissue macrophages

ATMs were isolated using the procedure described in detail previously^9^. CD3^+^ CD4^+^ Foxp3^−^ non-Treg T cells were isolated from the spleen of the Foxp3-EGFP mouse. 1 × 10^5^ non-Treg T cells were cocultured with 2 × 10^4^ ATMs in 200 μl of complete medium, 1 μg/ml of soluble anti-CD3e mAb (#1452C-11; BioLegend, San Diego, CA) and human 2 ng/ml rTGF-β (R&D Systems, Minneapolis, MN) in the presence of each fatty acid for 6 days. EPA was first dissolved in ethanol or added directly to the complete media. EPA metabolites were prepared by evaporating ethanol by N_2_ and dissolved directly in complete media. For induction of Treg T cells, 1 × 10^5^ non-Treg T cells (lacking ATMs) were incubated with human 1 ng/ml rTGF-β, 1000 units/ml IL-2 and 1:2 cell to beads ratio of CD3 and CD28 T activator dynabeads (#11452D, Thermo Fisher Scientific) or instead with dynabeads CD3 antibody (BioLegend) and CD28 antibody (BioLegend). Six days later, the proportion of Tregs was analyzed by FACSVerse (BD Biosciences, Franklin Lakes, NJ).

### Flow cytometry

Fluorescence-activated cell sorting (FACS) analysis was carried out as described in detail previously^9^. Briefly, cells in the SVF were suspended in FACS buffer and incubated with anti-mouse CD16/CD32 (#93; BioLegend) for 15 min. Then, the cells were rinsed and resuspended in FACS buffer and stained for 25 min with anti-CD45 (#30F-11; Biolegend), anti-CD11b (M1/70; BioLegend), anti-MHC-class II (#M5/114.15.2, eBioscience, San Diego, CA), anti-F4/80 (#BM8, Biolegend), CD11c (#N418; Biolegend) and anti-siglecF (#E50-2440, BD Pharmingen, San Diego, CA) for macrophages, dendritic cells and eosinophils. For B cells, CD4^+^ T cells, CD8^+^ T cells, natural killer (NK) cells, and NKT cells, SVF was incubated with anti-B220 (#RA3-6B2, Biolegend), anti-CD19 (#6D5, Biolegend), anti-NK1.1 (#PK136, Biolegend), anti-CD8 (#53–6.7, Biolegend), anti-CD4 (#RM4-5, Biolegend) and anti-CD3 (#17A2, Biolegend). For preadipocytes, endothelial cells and hematopoietic cells, SVF was incubated with anti-CD45 (#30F-11, Biolegend), anti-CD31 (# 390, Biolegend), anti-CD34 (#RAM34, eBioscience) and anti-PDGFRα (#APA5, Biolegend). After washing twice, the SVF was resuspended with 400 μl of FACS buffer, and 20 μl of precision count beads (Biolegend) was added as internal control and analyzed with FACSVerse (BD Biosciences). The absolute cell count was determined according to the instructions provided by the manufacturer.

### Intracellular staining

Freshly isolated cells were incubated with Fc Block for 25 min at 4 °C and then stained with anti-CD4 antibody (#GK1.5, BioLegend), anti-CD25 antibody (#PC61, Biolegend) and anti-CD3e antibody (#145-2C11, Biolegend). Intracellular staining of Foxp3 was conducted by using the Foxp3/transcription factor staining buffer set, according to the instructions provided by the manufacturer (eBioscience). For Foxp3 staining, Foxp3-antibody (#fjk-16s, eBioscience) was added, followed by additional double washing. The washed cells were analyzed with FACSVerse (BD Biosciences). Tregs were identified by gating on the CD4^+^ Foxp3^+^ cell population.

### Statistical analysis

All data were expressed as mean ± SEM. Differences between two groups were examined for statistical significance by the Student’s t-test. Bonferroni correction or Dunnett’s test was applied to the multiple comparisons. *P* value < 0.05 denoted the presence of a statistically significant difference.

## Results

### Dietary EPA increases the number of adipose tissue Tregs, eosinophils and B cells in normal chow-fed mice

**Figure 1:**
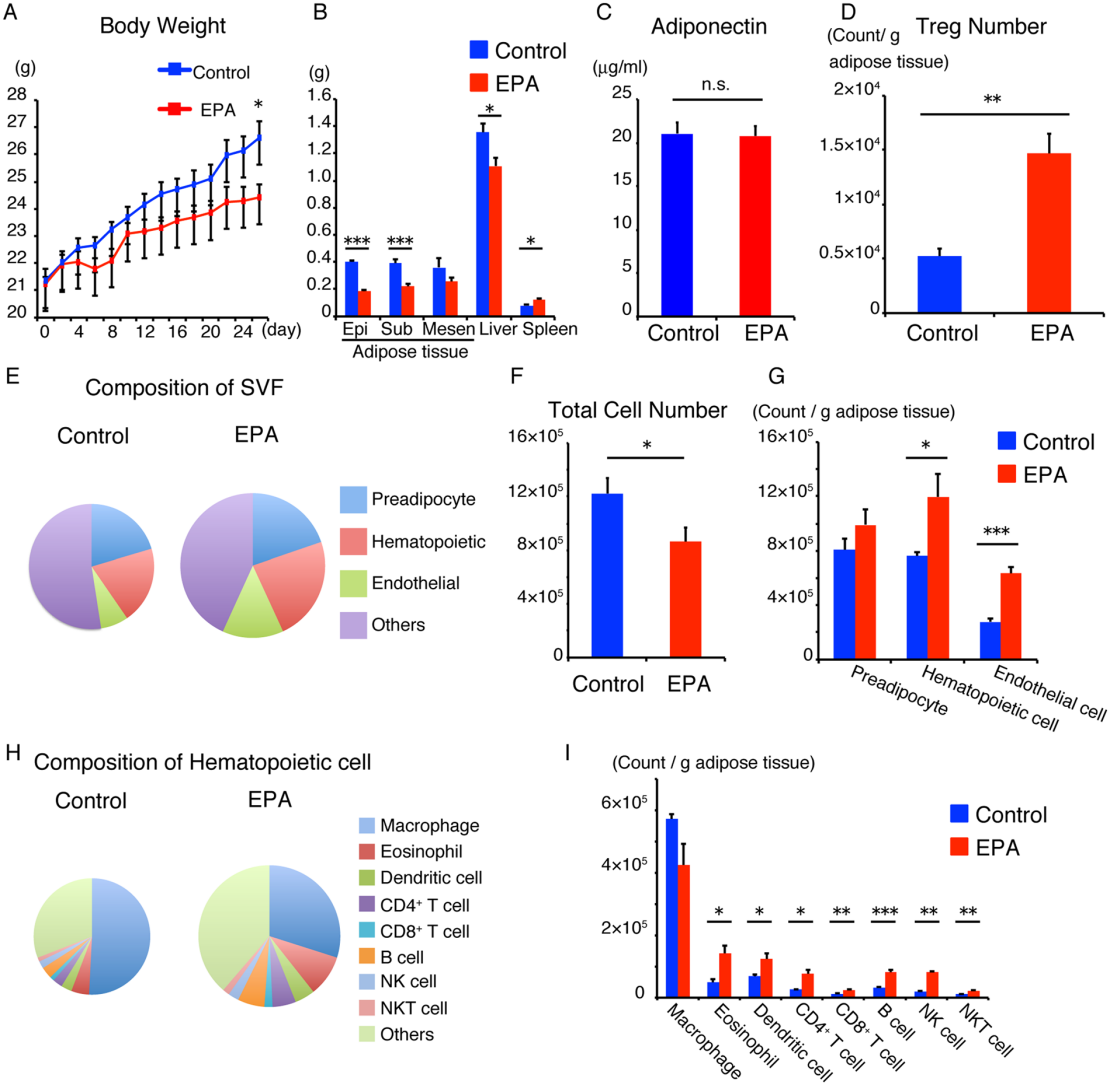
Dietary EPA increases the number of adipose tissue Tregs, eosinophils and B cells in normal chow-fed mice. C57BL/6J mice were fed normal diet free of fish oils or 5% EPA-containing diet for 4–5 weeks. (**A**) Body weight of C57BL/6J mice during treatment with 5% EPA starting from 5 weeks of age (n = 6). (**B**) Organ weight of C57BL/6J mice treated with 5% EPA (n = 6). (**C**) Serum adiponectin levels of mice fasted for 4 hours (n = 6). (**D**–**H**) Flow cytometric analysis of SVF of adipose tissues from 5% EPA-treated mice was performed. (**D**) Number of epididymal adipose tissue Treg per 1 g adipose tissue. (n = 6). (**E**) Amount of total cell number per g adipose tissue (area of circular chart) and composition of stromal vascular fraction from epididymal adipose tissue (% of total SVF). (**F**) Total cell number of SVF per adipose tissue (n = 8). (**G**) Number of preadipocytes, hematopoietic cells and endothelial cells (n = 6). (**H**) Amount of CD45^+^ immune cell number per g adipose tissue (area of circular chart) and composition of immune cells (% of CD45^+^ cells). (**I**) Number of adipose immune cells (n = 6). Data are mean ± SEM. *P < 0.05, **P < 0.01 and ***P < 0.001. Epi, epididymal adipose tissue: Sub, subcutaneous adipose tissue: Mesen, mesenteric adipose tissue.

Previous studies showed that EPA supplementation lowered body weight and improved hypoadiponectinemia in a high fat diet-fed model^19^. To determine the effect of EPA on adipose tissue immune cells under steady state conditions, mice were fed 5% EPA-containing diet for 5 weeks from 5 week of age and their adipose tissues were analyzed for changes in adipose immunocytes. EPA significantly lowered the body weight and weight of adipose tissues under steady state conditions (Fig. 1A and B). In contrast to EPA-fed obese model, there were no significant differences in serum adiponectin levels between EPA- and normal chow-fed wild-type mice (Fig. 1C).

Adipose tissue is composed of mature adipocytes and SVF, including preadipocytes (CD45^−^ CD31^−^ CD34^+^ PDGFRα^+^ cells), vascular endothelial cells (CD45^−^ CD31^+^ cells) and hematopoietic cells (CD45^+^ cells) (Supplementary Fig. 1A–C). Changes in SVF are considered to be important in chronic inflammation of the adipose tissue. To gain insight into the anti-inflammatory function of EPA toward adipose tissue, we examined the proportion and number of preadipocytes, endothelial cells and hematopoietic cells, including adipose tissue Tregs. The number of adipose tissue Tregs was almost three times higher in EPA-fed mice, relative to the control (Fig. 1D). The proportion and number of hematopoietic cells and endothelial cells were also significantly higher (Fig. 1E and G), possibly reflecting neoangiogenesis^28^ and increased infiltrating hematopoietic cells. While the total cell number per tissue was decreased (Fig. 1F), the cell number per adipose tissue weight exhibited the tendency to increase (Fig. 1E and G) reflecting the reduction of epididymal fat weight (Fig. 1B). We also examined the composition of hematopoietic cells (Fig. 1H and I). While the number of macrophages was not altered, anti-diabetic adipose tissue immune cells such as eosinophils, B cells and NKT cells were increased (Fig. 1I). Interestingly, even inflammatory dendritic cells and CD8^+^ T cells were also significantly higher in EPA-fed mice, relative to the control, because of the significant increase in the total amount of hematopoietic cells (Fig. 1H and I).

### Dietary EPA increase the number and proportion of Tregs regardless of the size of adipose tissue in ob/ob mice

**Figure 2:**
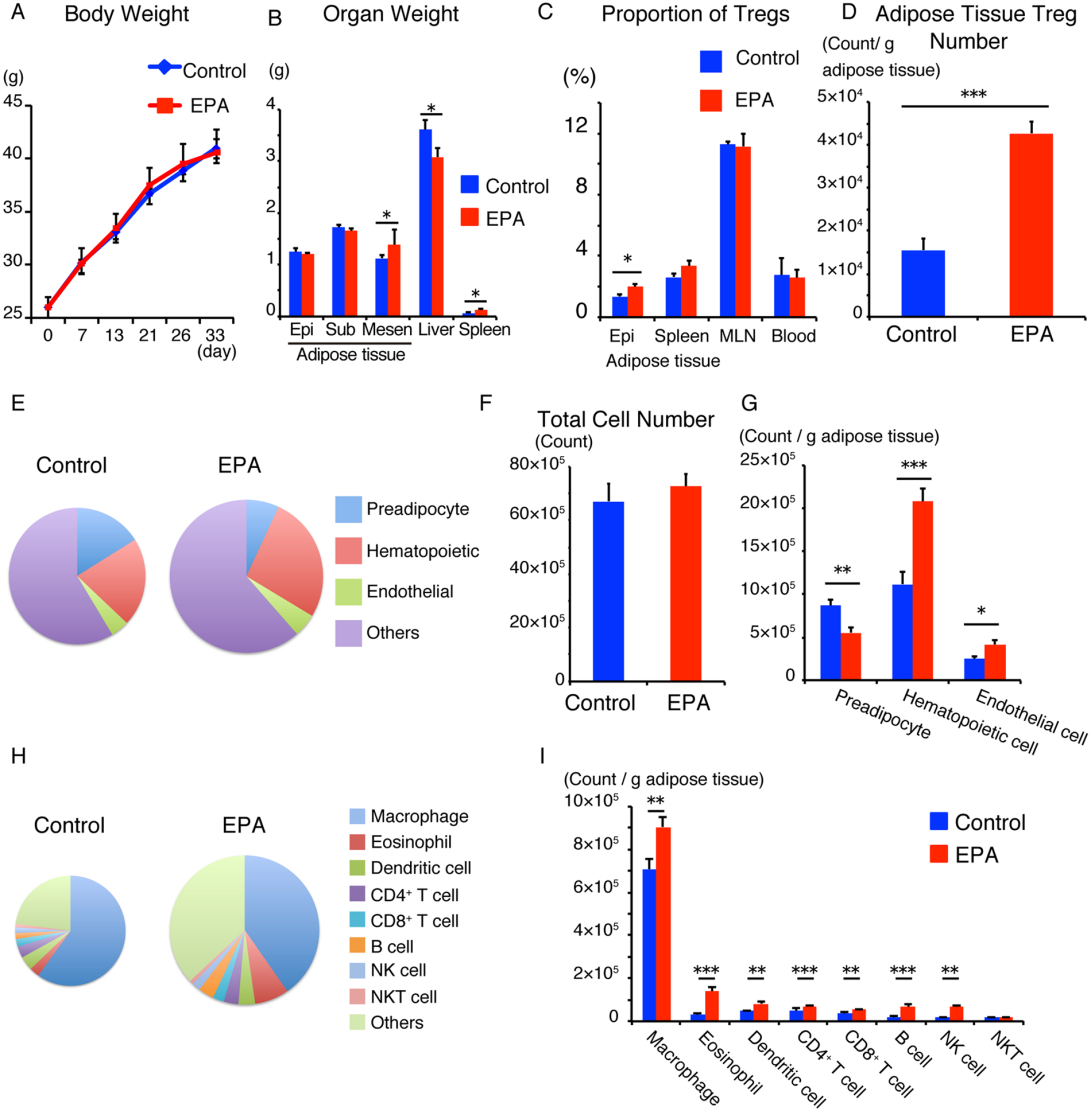
Dietary EPA increases the number and proportion of Tregs irrespective of the size of adipose tissue in ob/ob mice. ob/ob mice that lack leptin and express excess food intake and obesity were fed normal diet free of fish oils, or 5% EPA-containing diet for 4–5 weeks. (**A**) Body weight of ob/ob mice during treatment with 5% EPA starting from 5 weeks of age (n = 8). (**B**) Organ weight of ob/ob mice treated with 5% EPA (n = 8). (**C**) Serum adiponectin of mice fasted for 4 hours (n = 8). (**D**–**H**) Flow cytometric analysis of SVF of adipose tissues from 5% EPA-treated mice. (**D**) Number of Epididymal adipose tissue Treg per 1 g adipose tissue (n = 6). (**E**) Amount of total cell number per g adipose tissue (area of circular chart) and composition of stromal vascular fraction (% of total SVF). (**F**) Total cell number of SVF per tissue (n = 8). (**G**) Number of preadipocytes, hematopoietic cell and endothelial cells (n = 8). (**H**) Amount of CD45^+^ immune cell number per g adipose tissue (area of circular chart) and composition of immune cells (% of CD45^+^ cells). (**I**) Number of adipose immune cells (n = 8). Data are mean ± SEM. *P < 0.05, **P < 0.01 and ***P < 0.001. Epi, epididymal adipose tissue: Sub, subcutaneous adipose tissue: Mesen, mesenteric adipose tissue.

EPA has a different effect on body weight in ob/ob mice compared with high fat diet-fed mice. Previous studies reported that EPA treatment decreased the body weight of high fat-fed mice but had no such effect on that of ob/ob mice^19, 26^. In order to exclude the effect of loss of body weight on the composition of immune cells, we administered 5% EPA containing diet to ob/ob mice. In agreement with the results of previous studies, 5% EPA had no effects on the body weight and weight of epididymal adipose tissue (Fig. 2A and B). Interestingly, 5% EPA significantly increased the weight of mesenteric adipose tissue and spleen, while it significantly decreased that of the liver (Fig. 2B). The proportion of Tregs was elevated only in epididymal adipose tissue, but not in spleen, mesenteric lymph node (MLN), or blood (Fig. 2C). Congruent with the results of previous reports, the proportion of adipose tissue Tregs was relatively low in ob/ob mice, compared to C57BL/6J mice (ob/ob: 2–3%, C57BL/6J mice: 15%)^4^. Furthermore, the number of adipose tissue Treg was significantly higher in the adipose tissue of EPA-fed ob/ob mice, compared to the control (Fig. 2D). As was observed in C57BL/6J mice (Fig. 1E and F), the total numbers of CD45^+^ hematopoietic cells and vascular endothelial cells were significantly higher in EPA-fed ob/ob mice than the control (Fig. 2E and G). In contrast to C57BL/6J mice, the proportion and number of preadipocytes were lower in EPA fed ob/ob mice (Fig. 2E and G). In contrast to EPA-fed C57BL/6J mice, total cell number was unchanged in EPA-fed ob/ob mice (Fig. 2F).

Consistent with the results in C57BL/6J mice, the number of adipose immunocytes, including eosinophils, B cells, CD4^+^ T cells and CD8^+^ T cells, were significantly higher in EPA-fed mice. In contrast to C57BL/6 J mice, ATMs were significantly higher in EPA-fed ob/ob mice (Fig. 2H and I). These results indicate that EPA mainly increases the number of anti-inflammatory adipose immune cells regardless of body weight and adipose tissue size.

### EPA and its metabolite 5-HEPE up-regulate Treg induction in the presence of macrophages

**Figure 3:**
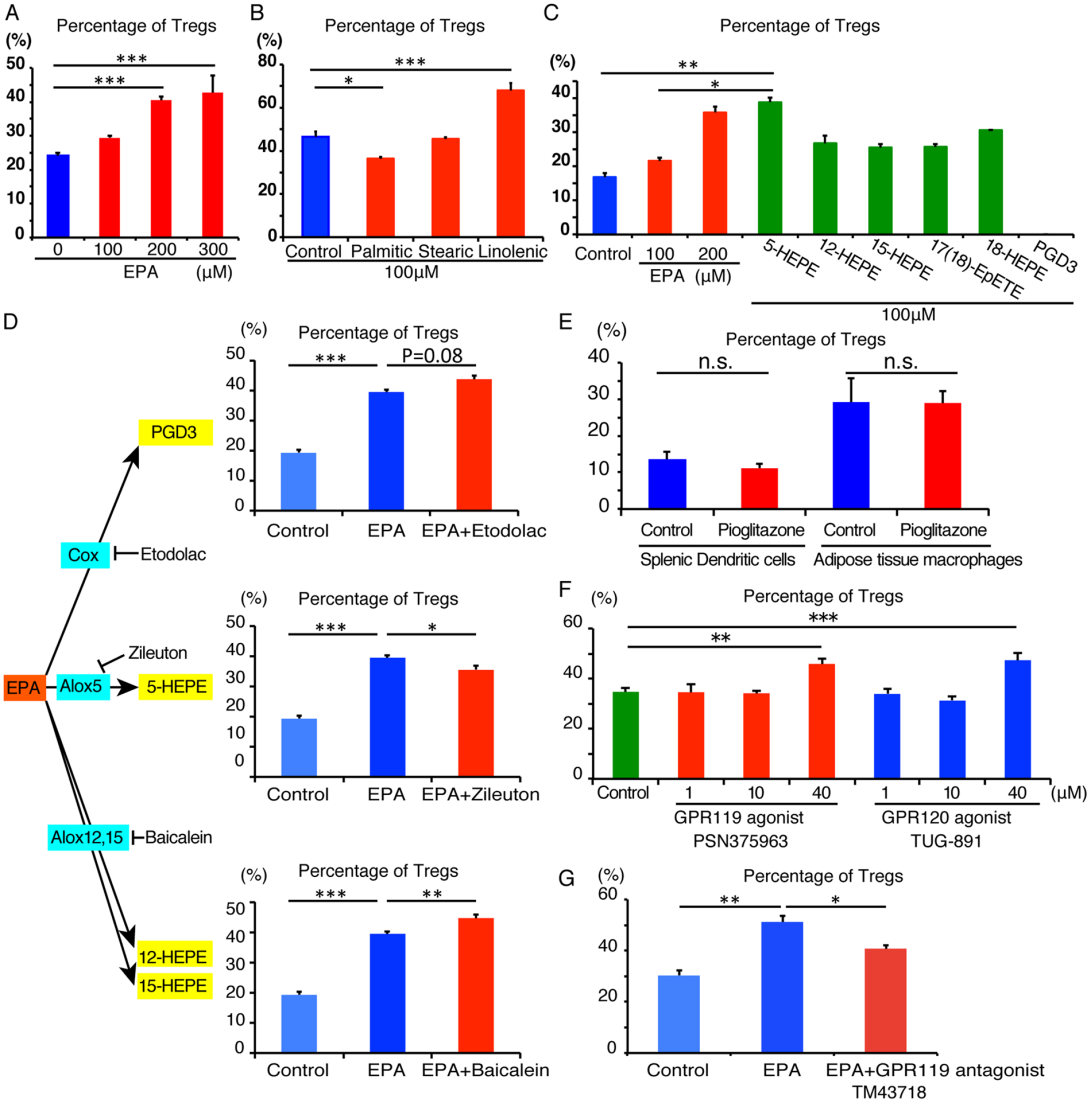
EPA and its metabolite 5-HEPE up-regulate Treg induction in the presence of macrophages. Effects of EPA, fatty acids and EPA metabolites on *in vitro* induction of Foxp3^+^ regulatory T cells stimulated with CD3ε antibody, adipose tissue macrophages, and TGFβ. Foxp3 expression was assessed by flow cytometry on day six of culture. (**A**) Effects and dose-dependency of EPA on *in vitro* Treg induction from Foxp3^−^ T cells isolated from Foxp3 EGFP mice. (**B**) Effects of the indicated fatty acids (100 μM) on *in vitro* Treg induction (n = 4). (**C**) Effects of the indicated purified EPA metabolites (100 μM) on *in vitro* Treg induction (n = 6). (**D**) Effects of pharmacological inhibition of the indicated EPA metabolic enzymes on *in vitro* Treg induction (n = 6). (**E**) The effect of 20 μM pioglitazone on in vitro Treg induction by splenic dendritic cells and adipose tissue macropahges (n = 3). (**F**) Isolated CD3^+^ CD4^+^ Foxp3^−^ T cells were co-cultured with adipose tissue macrophages in the presence of GPR119 agonist PSN375963 or GPR120 agonist TUG-891. Percentage of Tregs induced by adipose tissue macrophages in the presence of GPR119 or GPR120 agonist (n = 3). (**G**) Percentage of Tregs induced by adipose tissue macrophages in the presence of EPA with or without 10 μM GPR119 antagonist TM43718 (n = 3). Data are mean ± SEM. *P < 0.05, **P < 0.01 and ***P < 0.001.

To further investigate the mechanism through which EPA enhances Treg induction, we performed *in vitro* Treg induction using ATMs in the presence of fatty acids, such as saturated fatty acids and ω3 fatty acid, or EPA metabolites. In the presence of EPA, ATMs enhanced Treg induction in a dose-dependent manner (Fig. 3A). Among the dietary fatty acids, linolenic acid, an ω3 polyunsaturated fatty acid, increased Treg induction (Fig. 3B). EPA is metabolized into hydroxyl-EPA (HEPE) and/or pro-resolving lipid mediators (such as resolvin E1). EPA metabolism is dependent on four enzymatic pathways: cyclooxygenase (COX) 1/2, 5-arachidonate lipoxygenase (ALOX5), 12/15-arachidonate lipoxygenase (ALOX12/15) and cytochrome P450 (Cyp450). EPA is converted into prostaglandin D3 by COX1/2, 5-HEPE by ALOX5, 12-HEPE and 15-HEPE by ALOX12/15, and 17 (18)-EpETE by Cyp450. In addition to these enzymatic pathways, 18-HEPE is synthesized by a non-enzymatic pathway.

We hypothesized that the enhancement of Treg induction is dependent on one specific EPA metabolite because the effect of EPA is considered to be dependent on total functional effects of EPA itself and each EPA metabolite. To test our hypothesis, we performed Treg induction experiment in the presence of each EPA metabolite. Among EPA metabolites, 5-HEPE was the most potent inducer of Tregs (Fig. 3C). In order to confirm the role of 5-HEPE in the up-regulation of Treg induction, macrophages were co-cultured with non-Treg T cells in the presence of inhibitor for EPA metabolic genes (Fig. 3D). Inhibition of ALOX5, which is involved in the metabolism of EPA into 5-HEPE, reduced the EPA-dependent enhancement of Treg induction (Fig. 3D). Inhibition of cyclooxygenase or ALOX12,15 resulted in up-regulation of EPA-dependent enhancement of Treg induction (Fig. 3D).

EPA is a potent ligand for PPARγ and PPARγ agonist pioglitazone elevates the adipose tissue Tregs in high fat diet fed obese mice^29^. In order to elucidate whether the elevation of Treg induction by EPA depends on PPARγ, we co-cultured ATMs and naïve T cells in the presence of pioglitazone. Treg induction was not altered by pioglitazone (Fig. 3E), indicating that PPARγ is not involved in the enhancement of Treg induction by EPA.

5-HEPE is known as a potent ligand for GPR119^30^ and GPR120 is the receptor for long chain fatty acids, including EPA^20^. In order to examine whether the effect of EPA or 5-HEPE is dependent on GPR120 or GPR119, we performed co-culture experiments in the presence of GPR119 agonist PSN375963 or GPR120 agonist TUG-891 (Fig. 3F). Treg induction was enhanced in the presence of GPR119 agonist and GPR120 agonist, respectively (Fig. 3F). Moreover, Treg induction was blocked in the presence of TM43718, a specific antagonist of GPR119 (Fig. 3G), suggesting that enhancement of Treg induction by EPA and 5-HEPE is probably dependent on GPR119 signaling.

### ALOX5 inhibition reduces the effect of EPA on Treg maintenance *in viv*o

**Figure 4:**
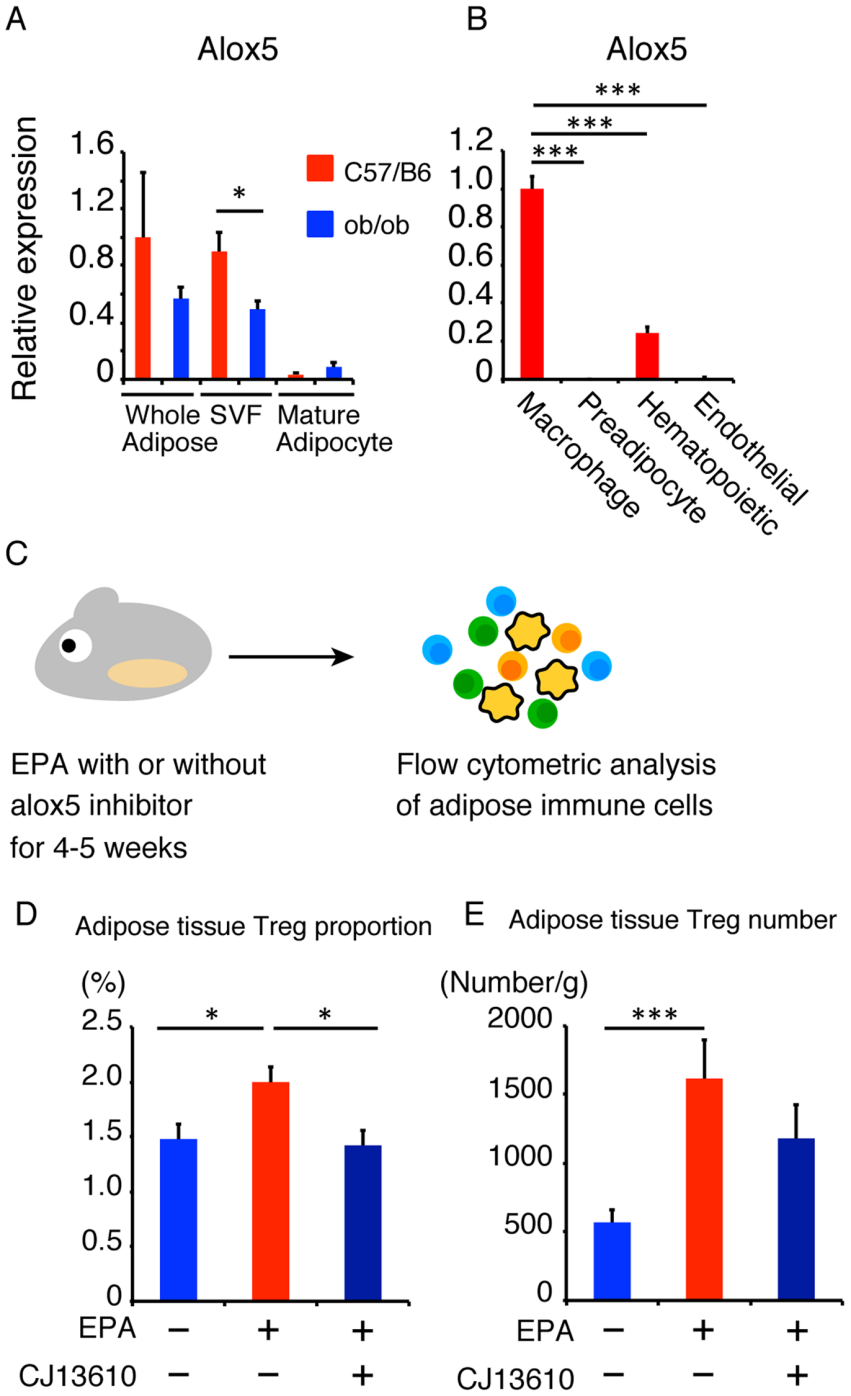
ALOX5 inhibition reduces the effect of EPA on Treg maintenance *in vivo*. (**A**) Expression of alox5 in primary adipose tissue, stromal vascular fraction and mature adipocytes. (**B**) Expression of alox5 in adipose tissue macrophages, preadipocytes, hematopoietic cells and endothelial cells isolated from adipose tissue stromal vascular fractions (n = 3). (**C**) ob/ob mice were fed 5% EPA-containing diet with or without ALOX5 inhibitor CJ13610 for 4–5 weeks. (**D**) FACS analysis of the proportion of adipose tissue Tregs (n = 4). (**E**) FACS analysis of the number of adipose tissue Tregs (n = 4). *P < 0.05, **P < 0.01 and ***P < 0.001.

To investigate the types of cells in which EPA is metabolized to 5-HEPE, we analyzed the expression of genes involved in EPA metabolism. We investigated the expression levels of these genes in primary adipose tissue, SVF and mature adipocytes, and compared the findings in C57BL/6J mice and ob/ob mice. The expression levels of EPA metabolic genes were significantly higher in SVF compared to mature adipocytes (Figs 4A and 2A). Furthermore, the expression of alox5 in SVF was significantly higher in C57BL/6J mice than in ob/ob mice (Fig. 4A). We next sorted SVF into macrophages (CD45^+^ CD11b^+^ F4/80^+^ subset), hematopoietic cells without macrophages (CD45^+^ subset − CD45^+^ CD11b^+^ F4/80^+^ subset), preadipocytes (CD45^−^ CD31^−^ CD34^+^ PDGFRα^+^ subset) and endothelial cells (CD45^−^ CD31^+^ subset) as shown in Supplementary Figure 1A–C. The expression levels of alox5, alox12 and cox1 were higher in macrophages (Figs 4B and 2B). The expression level of COX2 was higher in preadipocytes, while the expression level of alox12 was higher in macrophages and endothelial cells (Supplementary Fig. 2B). These results indicate that the conversion of EPA into 5-HEPE seems to occur in macrophages.

To further confirm the role of 5-HEPE in Treg induction *in vivo*, mice were treated with EPA and CJ13610, an ALOX5 inhibitor, for 4–5 weeks and then analyzed the changes in adipose tissue immune cells (Fig. 4C). Previous reports indicated that inhibition of ALOX5 down-regulated genes associated with hepatic fatty acid uptake, such as L-FABP and CD36 and improves fatty liver in ob/ob mice^31^. Administration of EPA with CJ13610 significantly decreased the proportion of adipose tissue Tregs (Fig. 4D). The number of adipose tissue Tregs also tended to be lower, compared to ob/ob mice fed EPA alone (Fig. 4E). These data suggest that ALOX5 and 5-HEPE seem to contribute to adipose tissue Treg induction *in vivo*.

### Pretreatment with EPA or Treg induction without macrophages does not enhance Treg induction

**Figure 5:**
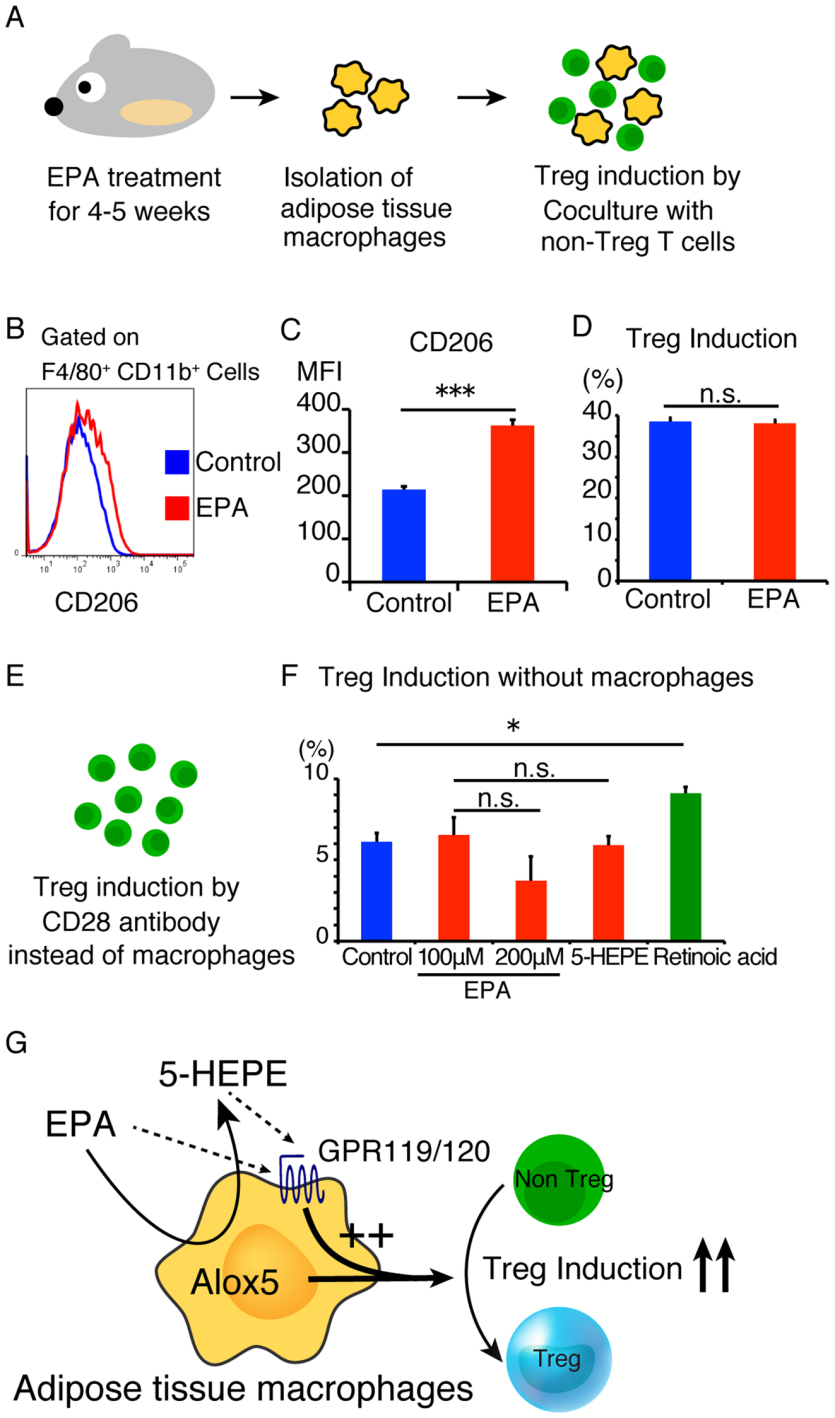
Pretreatment with EPA or Treg induction in the absence of macrophages did not increase Treg induction. (**A**) C57BL/6 J mice were fed 5% EPA-containing diet for 4–5 weeks followed by isolation of adipose tissue macrophages. The isolated macrophages were co-cultured with Foxp3^−^ T cells for 6 days. (**B**) (**C**) Flow cytometric analysis of CD206 expression on F4/80^+^ CD11b^+^ macrophages. (**D**) Percentage of Foxp3^+^ Tregs induced by adipose tissue macrophages isolated from EPA-fed mice (n = 4). (**E**) Isolated CD3^+^ CD4^+^ Foxp3^−^ T cells were stimulated with CD3ε antibody, CD28 antibody (or anti-CD3/28 coated beads), IL-2 and TGFβ in the absence of macrophages. (**F**) Percentage of Tregs induced in the presence of the indicated fatty acids in the absence of macrophages. Retinoic acid was used as positive control (n = 3). Data are mean ± SEM. **P < 0.01 and ***P < 0.001. (**G**) Proposed model for cellular events involved in the enhancement of Treg induction by EPA and 5-HEPE.

To elucidate the mechanisms of Treg induction, especially the target cells of EPA, we co-cultured ATMs derived from EPA-fed mice with non Treg T cells (Fig. 5A). ATMs expressed higher levels of CD206, a marker of M2 macrophages, indicating that they polarized towards the anti-inflammatory M2 phenotype (Fig. 5B and C). However, the co-culture was not associated with a change in the proportion of induced Tregs (Fig. 5D), suggesting that M2 polarization by EPA did not up-regulate Treg induction.

To determine whether EPA directly enhances Treg induction, we induced Tregs in the presence of CD3 antibody, CD28 antibody, IL-2 and TGF-β (Fig. 5E). In the absence of macrophages, Treg induction was not enhanced in the presence of EPA or 5-HEPE (Fig. 5F), suggesting that EPA and 5-HEPE enhance Treg induction via macrophages.

## Discussion

There is general agreement that EPA has pleiotropic and anti-inflammatory effects on various tissues and lesions such as atherosclerotic lesions^32^, hepatic steatosis^33^ and adipose tissues^34^. While chronic inflammation of the adipose tissue involves increased number of inflammatory immune cells, such as M1 polarized macrophages and CD8^+^ T cells, and reduction of anti-inflammatory immune cells, such as Tregs, eosinophils and B cells, it is still not clear how EPA improves chronic inflammation in adipose tissue. Here, we show that both EPA and its metabolite 5-HEPE enhance ATM-mediated Treg induction.

We reported previously that ATMs induce PPARγ^+^ Tregs in adipose tissues^9^. Considering that supplementation with ω3 fatty acid inhibit polarization of inflammatory Th17 T cells^35^ and M1 macrophages^20^, we hypothesized that EPA could improve adipose tissue inflammation via enhancement of Treg induction by M2-polarized macrophages. Consistent with this hypothesis, the present results of flow cytometric analysis showed increased number of adipose tissue Tregs irrespective of adiposity. In our previous report, ATMs induced Tregs in adipose tissues, and the proportions of ATMs and adipose tissue Tregs were lower in adiponectin-deficient mice than control mice^9^. Since EPA supplementation increased serum adiponectin levels in ob/ob mice, one possible explanation of the induction of ATMs and adipose tissue Tregs is upregulation of serum adiponectin levels (Supplementary Fig. 5).

EPA reduces weight gain by elevation of oxygen consumption, and UCP1 expression in both interscapular BAT and inguinal WAT^36^ without any changes in food intake. On the other hand, Obesity strikingly reduces the number of CD4^+^ Foxp3^+^ Treg cells in adipose tissues^4^. Therefore, weigh-loss itself is partly responsible for the induction of adipose tissue Tregs by EPA in C57BL/6J mice. To exclude the effect of EPA to reduce body weight, we treated ob/ob mice with EPA, and revealed that the number of adipose tissue Treg was significantly higher in EPA-treated mice compared to control. Our results indicated that the effect of EPA to induce Tregs is independent of weight loss.

The molecular mechanisms by which EPA lowers body weight and improves glucose metabolism is mainly dependent on activation of PPARγ^37^ and GPR120^20^. Among adipose tissue immune cells, macrophages and Tregs have been reported to express PPARγ, raising the possibility that the effect of EPA on adipose tissue immune cells is dependent on PPARγ. Under our experimental condition, pioglitazone, a PPARγ agonist, had no significant impact on Treg induction by spleen dendritic cells or ATMs (Fig. 3E). These results indicated that PPARγ pathway does not play a large role in EPA-mediated Treg induction.

5-HEPE is the major metabolite in macrophage conditioned media^22^. It is also the endogenous agonist for GPR119 and increases insulin secretion from Min6 cells in GPR119-dependent manner^30^. Although high serum 5-HEPE levels have been described in overweight subjects following polyunsaturated fatty acid supplementation^38^, the physiological function of 5-HEPE in adipose tissue has not been elucidated. We demonstrated here that both 5-HEPE and synthetic GPR119 agonist enhanced the induction of Tregs by adipose tissue macrophages. Moreover, antagonist of GPR119 strongly blocked macrophage-dependent Treg induction by EPA. Considering that 5-HEPE is a specific ligand for GPR119^39^, we concluded that EPA induced Treg induction mainly through its metabolite, 5-HEPE via GPR119.

Previous studies reported that IL-2, IL-33^40^, retinoic acid^41^ and butyrate^42^ act directly on T lymphocytes and increase the induction of Tregs but not macrophages. In the present study, EPA and 5-HEPE enhanced Treg induction in the presence of macrophages, but not in their absence. These results suggest that EPA and 5-HEPE activate signaling pathways in macrophages. Next, we investigated the effects of EPA on macrophages *in vivo*, and demonstrated that ATMs of EPA-fed mice expressed high levels of CD206, a marker of M2 macrophages, indicating that EPA induces polarization of ATMs into anti-inflammatory M2 phenotype. We reported previously that M1 polarized ATMs from obese adipose tissue induced almost equivalent proportion of Tregs compared to macrophages derived from lean mice model^9^. Similarly, in the present study, M2 polarized macrophages from EPA-fed mice induced equivalent proportion of Tregs compared to normal chow-fed mice. These results suggest that it is important for enhanced Treg induction to activate GPR119 signaling by EPA or 5-HEPE in macrophages during, but not prior to co-culture. Furthermore, the gene expression levels of GPR119 and GPR120 were quite low in T cells (data not shown), supporting the direct effects of 5-HEPE and EPA on macrophages.

In addition, we confirmed the direct effect of EPA and 5-HEPE on macrophages by treatment with EPA and 5-HEPE, which reduced the expression levels of IL-1β, iNOS, or MCP1 in RAW264.7 macrophages (Supplementary Fig. 3A). On the other hand, the expressions of genes that are associated with Treg induction and function such as TGFβ, indoleamine-2,3-deoxygenase (IDO) and retinal dehydrogenase (RALDH) were low in ATMs (Supplementary Fig. 3B). Also, EPA and 5-HEPE decreased the expression of these genes, indicating that IDO, RALDH and TGFβ are not involved in the enchancement of ATM mediated Treg induction by EPA (Supplementary Fig. 3C). Because such genes related to Treg induction were not changed by EPA or 5-HEPE, further investigation will be required to identify genes associated with macrophage-mediated Treg induction.

Interestingly, the phenomena of ATM mediated Treg induction by EPA and 5-HEPE is also observed when ATMs are replaced by splenic dendritic cells (Supplementary Fig. 4A and B), suggesting the direct effect of EPA and 5-HEPE on general antigen presenting cells (Supplementary Fig. 4A and B).

The number of preadipocytes were reduced by EPA in ob/ob mice, but not in C57BL/6J mice. One possible explanation for the reduction of preadipocytes is growth arrest or apoptosis of preadipocytes in adipose tissues of ob/ob mice. In human preadipocytes cell line AML-I, EPA induces growth arrest and apoptosis, whereas it promotes differentiation into adipocyte^43^. Moreover, high glucose concentration also induces premature senescence, apoptosis and reduced colony forming activity in mesenchymal stem cells^44^. Collectively, combination of EPA and high-glucose levels might be responsible for the smaller number of preadipocytes in ob/ob mice, but not in C57BL/6J mice.

EPA exhibits anti-inflammatory properties through its metabolites in each organ such as heart^22^, skin^23^ and gut^45^. Our data suggest that the enzymes associated with EPA metabolism are expressed mainly in SVF of adipose tissues and are downregulated in obesity. ALOX5 is involved in the generation of 5-HEPE, 5-oxo-EPE, leukotriene A5, and leukotriene B5. When acting with other lipoxygenases, cyclooxygenases, or cytochrome P450 enzymes, ALOX5 contributes to synthesizing resolvin Es, which is a potent anti-inflammatory lipid mediator derived from EPA^46^. ALOX5 is expressed mainly in macrophages, indicating that the conversion of EPA into 5-HEPE is processed mainly in macrophages. Our results showed that inhibition of ALOX5 reduced the proportion of Tregs both *in vitro* and *in vivo*, suggesting the importance of EPA metabolites of ALOX5, such as 5-HEPE and resolvinEs. Given that resolvin Es is generated from 18-HEPE and the addition of 18-HEPE did not induce Tregs compared to 5-HEPE, reduced Treg induction is likely to be attributed to the decrease in 5-HEPE by ALOX5 inhibition.

These findings lead us to propose a model in which EPA enhances the induction of adipose tissue Tregs through its metabolite, 5-HEPE, and functional involvement of GPR120 and GPR119 (Fig. 5G). The pleiotropic effects of EPA probably involve each metabolite; which serve as ligands for different receptors. Further elucidation of the effect and the target of EPA metabolites can help in the design of specific treatments for various metabolic diseases.

## Electronic supplementary material

supplementary information

## References

[1] Xu H (2003). Chronic inflammation in fat plays a crucial role in the development of obesity-related insulin resistance. J Clin Invest.

[2] Fantuzzi, G. Adipose tissue, adipokines, and inflammation. *J Allergy Clin Immunol* **115**, 911–919; quiz 920 (2005). 10.1016/j.jaci.2005.02.023 15867843

[3] Kawasaki N, Asada R, Saito A, Kanemoto S, Imaizumi K (2012). Obesity-induced endoplasmic reticulum stress causes chronic inflammation in adipose tissue. Sci Rep.

[4] Feuerer M (2009). Lean, but not obese, fat is enriched for a unique population of regulatory T cells that affect metabolic parameters. Nat Med.

[5] Nishimura S (2009). CD8+ effector T cells contribute to macrophage recruitment and adipose tissue inflammation in obesity. Nat Med.

[6] Doganci A (2005). The IL-6R alpha chain controls lung CD4 + CD25+ Treg development and function during allergic airway inflammation *in vivo*. J Clin Invest.

[7] Oldenhove G (2009). Decrease of Foxp3+ Treg cell number and acquisition of effector cell phenotype during lethal infection. Immunity.

[8] Nishikawa H, Sakaguchi S (2010). Regulatory T cells in tumor immunity. Int J Cancer.

[9] Onodera T (2015). Adipose tissue macrophages induce PPARgamma-high FOXP3(+) regulatory T cells. Sci Rep.

[10] Baer DJ, Judd JT, Clevidence BA, Tracy RP (2004). Dietary fatty acids affect plasma markers of inflammation in healthy men fed controlled diets: a randomized crossover study. Am J Clin Nutr.

[11] Kennedy A, Martinez K, Chuang CC, LaPoint K, McIntosh M (2009). Saturated fatty acid-mediated inflammation and insulin resistance in adipose tissue: mechanisms of action and implications. J Nutr.

[12] Poirier H, Shapiro JS, Kim RJ, Lazar MA (2006). Nutritional supplementation with trans-10, cis-12-conjugated linoleic acid induces inflammation of white adipose tissue. Diabetes.

[13] Miles EA, Calder PC (2012). Influence of marine n-3 polyunsaturated fatty acids on immune function and a systematic review of their effects on clinical outcomes in rheumatoid arthritis. Br J Nutr.

[14] Li J (2013). Intakes of long-chain omega-3 (n-3) PUFAs and fish in relation to incidence of asthma among American young adults: the CARDIA study. Am J Clin Nutr.

[15] Calder PC (2008). Polyunsaturated fatty acids, inflammatory processes and inflammatory bowel diseases. Mol Nutr Food Res.

[16] Zeyda M (2005). Polyunsaturated fatty acids block dendritic cell activation and function independently of NF-kappaB activation. J Biol Chem.

[17] Iwami D (2009). Purified eicosapentaenoic acid induces prolonged survival of cardiac allografts and generates regulatory T cells. Am J Transplant.

[18] Todoric J (2006). Adipose tissue inflammation induced by high-fat diet in obese diabetic mice is prevented by n-3 polyunsaturated fatty acids. Diabetologia.

[19] Kalupahana NS (2010). Eicosapentaenoic acid prevents and reverses insulin resistance in high-fat diet-induced obese mice via modulation of adipose tissue inflammation. J Nutr.

[20] Oh DY (2010). GPR120 is an omega-3 fatty acid receptor mediating potent anti-inflammatory and insulin-sensitizing effects. Cell.

[21] Xue B, Yang Z, Wang X, Shi H (2012). Omega-3 polyunsaturated fatty acids antagonize macrophage inflammation via activation of AMPK/SIRT1 pathway. PLoS One.

[22] Endo J (2014). 18-HEPE, an n-3 fatty acid metabolite released by macrophages, prevents pressure overload-induced maladaptive cardiac remodeling. J Exp Med.

[23] Sawada Y (2015). Resolvin E1 inhibits dendritic cell migration in the skin and attenuates contact hypersensitivity responses. J Exp Med.

[24] Chu ZL (2007). A role for beta-cell-expressed G protein-coupled receptor 119 in glycemic control by enhancing glucose-dependent insulin release. Endocrinology.

[25] Lauffer LM, Iakoubov R, Brubaker PL (2009). GPR119 is essential for oleoylethanolamide-induced glucagon-like peptide-1 secretion from the intestinal enteroendocrine L-cell. Diabetes.

[26] Itoh M (2007). Increased adiponectin secretion by highly purified eicosapentaenoic acid in rodent models of obesity and human obese subjects. Arterioscler Thromb Vasc Biol.

[27] Mita T (2007). Eicosapentaenoic acid reduces the progression of carotid intima-media thickness in patients with type 2 diabetes. Atherosclerosis.

[28] Nishimura S (2007). Adipogenesis in obesity requires close interplay between differentiating adipocytes, stromal cells, and blood vessels. Diabetes.

[29] Cipolletta D (2012). PPAR-gamma is a major driver of the accumulation and phenotype of adipose tissue Treg cells. Nature.

[30] Hansen HS, Rosenkilde MM, Holst JJ, Schwartz TW (2012). GPR119 as a fat sensor. Trends Pharmacol Sci.

[31] Lopez-Parra M (2008). Regulatory effects of arachidonate 5-lipoxygenase on hepatic microsomal TG transfer protein activity and VLDL-triglyceride and apoB secretion in obese mice. J Lipid Res.

[32] Liu L (2016). Protective role of n6/n3 PUFA supplementation with varying DHA/EPA ratios against atherosclerosis in mice. J Nutr Biochem.

[33] Chouinard-Watkins R (2016). A Diet Rich in Docosahexaenoic Acid Restores Liver Arachidonic Acid and Docosahexaenoic Acid Concentrations in Mice Homozygous for the Human Apolipoprotein E epsilon4 Allele. J Nutr.

[34] Flachs P (2006). Polyunsaturated fatty acids of marine origin induce adiponectin in mice fed a high-fat diet. Diabetologia.

[35] Monk JM, Hou TY, Turk HF, McMurray DN, Chapkin RS (2013). n3 PUFAs reduce mouse CD4+ T-cell *ex vivo* polarization into Th17 cells. J Nutr.

[36] Kim M (2015). Fish oil intake induces UCP1 upregulation in brown and white adipose tissue via the sympathetic nervous system. Sci Rep.

[37] Li H (2005). EPA and DHA reduce LPS-induced inflammation responses in HK-2 cells: evidence for a PPAR-gamma-dependent mechanism. Kidney Int.

[38] Nielsen MS (2012). The effect of low-dose marine n-3 fatty acids on the biosynthesis of pro-inflammatory 5-lipoxygenase pathway metabolites in overweight subjects: a randomized controlled trial. Prostaglandins Leukot Essent Fatty Acids.

[39] Kogure R, Toyama K, Hiyamuta S, Kojima I, Takeda S (2011). 5-Hydroxy-eicosapentaenoic acid is an endogenous GPR119 agonist and enhances glucose-dependent insulin secretion. Biochem Biophys Res Commun.

[40] Vasanthakumar A (2015). The transcriptional regulators IRF4, BATF and IL-33 orchestrate development and maintenance of adipose tissue-resident regulatory T cells. Nat Immunol.

[41] Sun CM (2007). Small intestine lamina propria dendritic cells promote de novo generation of Foxp3 T reg cells via retinoic acid. J Exp Med.

[42] Arpaia N (2013). Metabolites produced by commensal bacteria promote peripheral regulatory T-cell generation. Nature.

[43] Hanada H, Morikawa K, Hirota K, Nonaka M, Umehara Y (2011). Induction of apoptosis and lipogenesis in human preadipocyte cell line by n-3 PUFAs. Cell Biol Int.

[44] Khan M, Akhtar S, Mohsin S, S. NK, Riazuddin S (2011). Growth factor preconditioning increases the function of diabetes-impaired mesenchymal stem cells. Stem Cells Dev.

[45] Kunisawa J (2015). Dietary omega3 fatty acid exerts anti-allergic effect through the conversion to 17,18-epoxyeicosatetraenoic acid in the gut. Sci Rep.

[46] Schwab JM, Chiang N, Arita M, Serhan CN (2007). Resolvin E1 and protectin D1 activate inflammation-resolution programmes. Nature.

