# Supplementary material for: Eicosapentaenoic acid and 5-HEPE enhance macrophage-mediated Treg induction in mice
Source: Sci Rep. 2017 Jul 4;7:4560. doi: 10.1038/s41598-017-04474-2 (PMC5496870; doi:10.1038/s41598-017-04474-2)
Supplement: Supplementary file 1 — supplementary information [file 41598_2017_4474_MOESM1_ESM.pdf]

Eicosapentaenoic acid and 5-HEPE enhance macrophage-mediated Treg induction in mice

Toshiharu Onodera [a,b], Atsunori Fukuhara [a], Jihoon Shin [a], Tomonori Hayakawa [a], Michio Otsuki [a], Iichiro Shimomura [a]

[a] Department of Metabolic Medicine, Osaka University Graduate School of Medicine, 2-2, Yamamdaoka, Suita, Osaka, Japan

[b] Department of Diabetes Care Medicine, Osaka University Graduate School of Medicine, 2-2, Yamamdaoka, Suita, Osaka, Japan

Corresponding author:

Atsunori Fukuhara, M.D., Ph.D.

Department of Metabolic Medicine, Graduate School of Medicine, Osaka University

2-2 Yamadaoka, Suita, Osaka 565-0871, JAPAN

Tel: +81-6-6879-3732, Fax: +81-6-6879-3739

E-mail: fukuhara@endmet.med.osaka-u.ac.jp

Supplementary Table 1. Sequences of primers used in this study.

Gene		sequence
36B4	Forward	GCTCCAAGCAGATGCAGCA
	Reverse	CCGGATGTGAGGCAGCAG
IL-1β	Forward	TCGCTCAGGGTCACAAGAAA
	Reverse	CCATCAGAGGCAAGGAGGAA
iNOS	Forward	CAGCTGGGCTGTACAAACCTT
	Reverse	CATTGGAAGTGAAGCGTTTCG
IL-6	Forward	ACAACCACGGCCTTCCCTACTT
	Reverse	CACGATTTCCCAGAGAACATGTG
Cox1	Forward	TTCTGCCCTCTGTACCCAAA
	Reverse	AAGGATGAGGCGAGTGGTCT
Cox2	Forward	CCGTGGGGAATGTATGAGCA
	Reverse	CCAGGTCCTCGCTTATGATCTG
Alox5	Forward	ATTGCCATCCAGCTCAACCA
	Reverse	GCAGAAGGTGGGTGATCGTT
Alox12	Forward	GTTTGACTTCGACGTTCCCG
	Reverse	CGTCATCCACAACTGTGTGC
Alox15	Forward	GCGACGCTGCCCAATCCTAATC
	Reverse	ATATGGCCACGCTGTTTTCTACC
IDO	Forward	CTGCACGACATAGCTACCAGT
	Reverse	GCACTGCCCCCTGAAAACAT
TGFβ	Forward	ACCATGCCAACTTCTGTCTG
	Reverse	CGGGTTGTGTTGGTTGTAGA
RALDH1	Forward	CGCATTGCCAAAGAGGAGATAT
	Reverse	GAGTCCTGCTGCTAAACCATAGG
RALDH2	Forward	GCCCCTTTGATCCCACAACT
	Reverse	CACCGCTCTGGATAAGCTCC
RALDH3	Forward	GGGTTCTTGCCTCCTAGCTC
	Reverse	AAGATAGCCTTCACCGGCTC

36B4, ribosomal protein, large, P0 (Rplp0) ; IL-1β, Interleukin-1beta ; iNOS, Nitric Oxide Synthase Type 2 ; IL-6, Interleukin-6 ; Cox1, Cyclooxygenase 1 ; Cox2, Cyclooxygenase 2; Alox5, arachidonate 5-lipoxygenase ; Alox12, arachidonate 12-lipoxygenase ; Alox15, arachidonate 15-lipoxygenase ; IDO, indoleamine 2,3-dioxygenase ; RALDH1, aldehyde dehydrogenase1 ; RALDH2, aldehyde dehydrogenase2 ; RALDH3, aldehyde dehydrogenase3

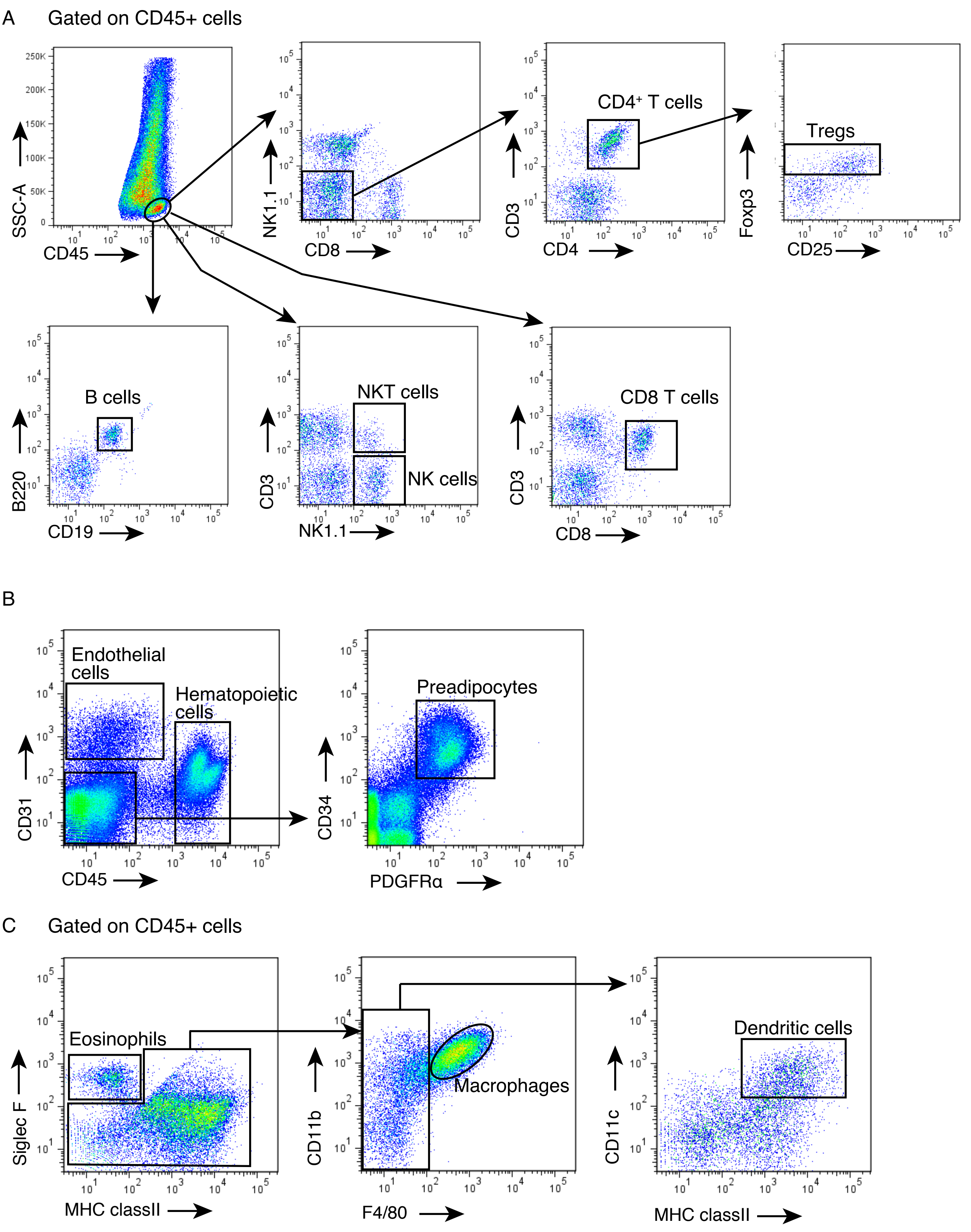

Supplementary figure 1 Gating strategies and dot plot images of SVF cells from epididymal adipose tissue.

(A) Lymphocytes of SVF from epididymal adipose tissue were stained for CD3, CD4, CD8, CD19, CD25, CD45, B220, NK1.1 and Foxp3. Each type of cells were defined as follows. (CD4$^+$ T cells : CD45$^+$CD3$^+$CD4$^+$ cells, CD8$^+$ T cells : CD45$^+$CD3$^+$ CD8$^+$ cells, Tregs : CD45$^+$CD3$^+$CD4$^+$Foxp3$^+$ cells, B cells : CD45$^+$CD19$^+$B220$^+$ cell, NK cells : CD45$^+$ CD3$^-$NK1.1$^+$ cells) (B) SVF from epididymal adipose tissue were stained for CD45, CD31, CD34 and PDGFRα. Endothelial cells, hematopoietic cells and preadipocytes were defined as follows. (Endothelial cells : CD45$^-$CD31$^+$ cells, Hematopoietic cells : CD45$^+$ cells, Preadipocytes : CD31$^-$CD45$^-$CD34$^+$PDGFRα$^+$ cells)
(C) Antigen presenting cells of SVFs from epididymal adipose tissue were stained for CD11b, CD11c, F4/80, SiglecF and MHC classII. Each type of antigen presenting cell is defined as follows. (Macrophages : CD45$^+$CD11b$^+$F4/80$^+$ cells, Dendritic cells : CD45$^+$SiglecF$^-$F4/80$^-$CD11c$^+$MHCclassII$^+$ cells, Eosinophils : CD45$^+$CD11b$^+$SiglecF$^+$ cells)

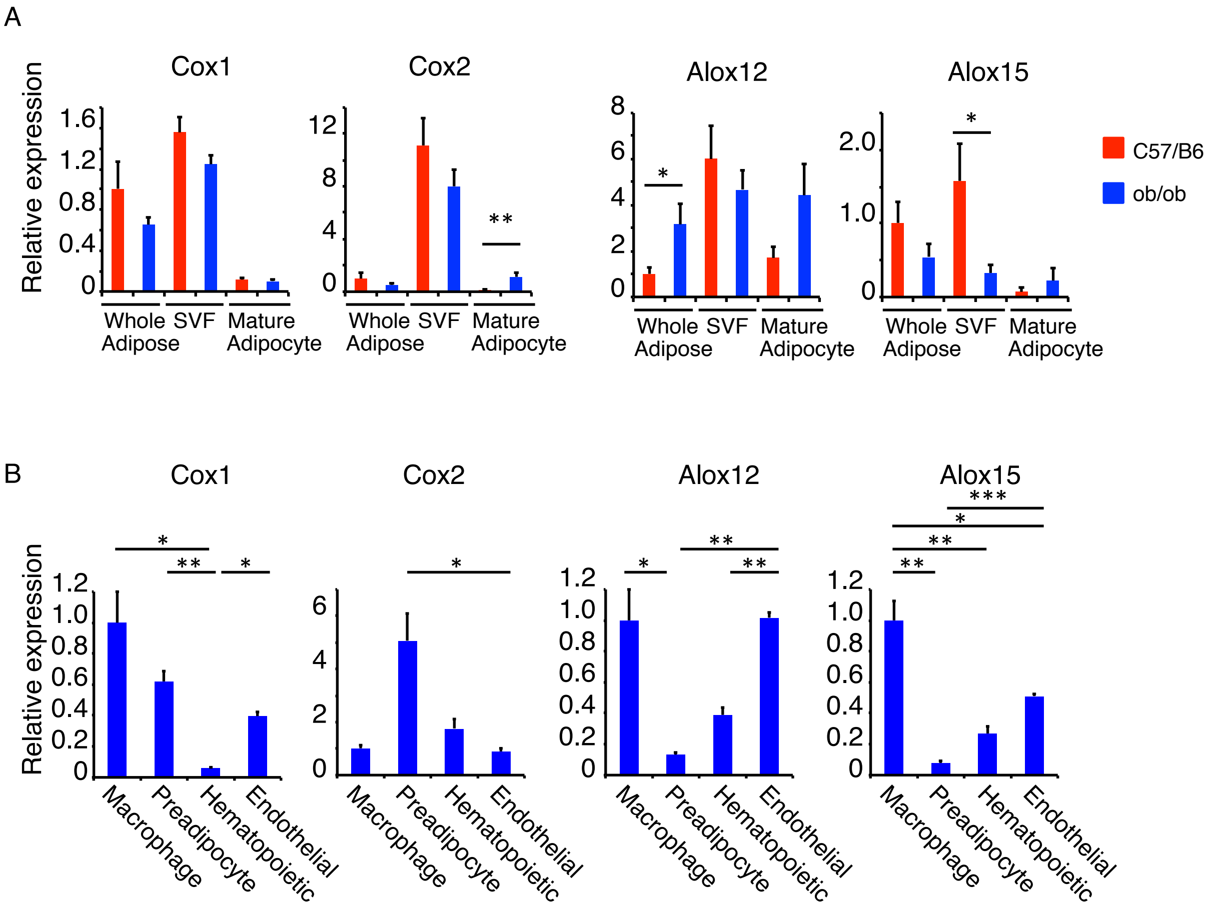

Supplementary figure 2 Localization and expression of genes that is involved in the EPA metabolism.

(A) Expression of genes associated with EPA metabolism in primary adipose tissue, stromal vascular fraction and mature adipocyte. (B) Expression of genes involved in EPA metabolism in adipose tissue macrophages, preadipocyte, hematopoietic cells and endothelial cells isolated from adipose tissue stromal vascular fractions. (n=3)

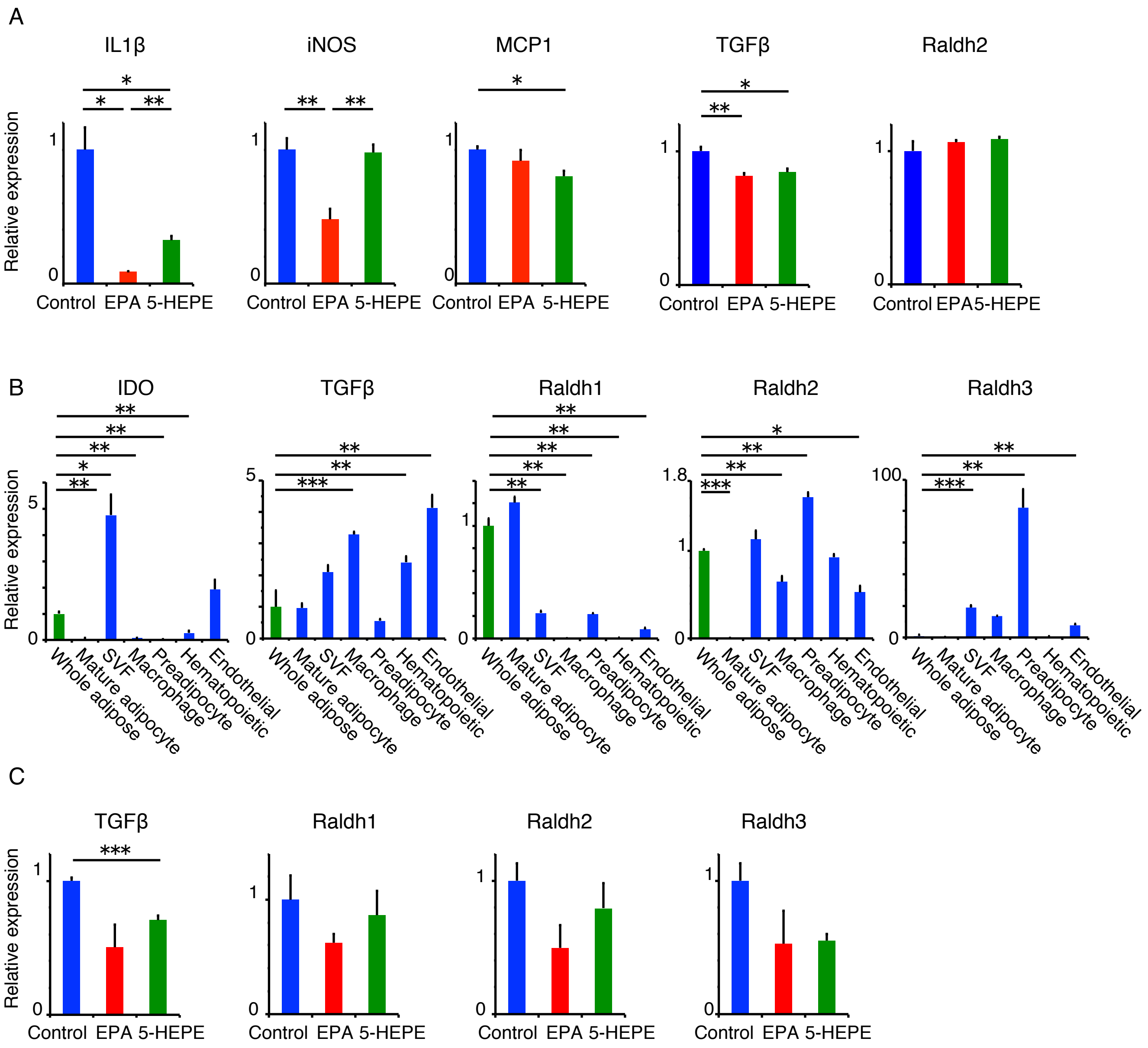

Supplementary figure 3 Effect of EPA and 5-HEPE on RAW264.7 and primary adipose tissue macrophages

(A) RAW264.7 macrophages were stimulated with 100μM EPA or 5-HEPE for 24 hours followed by measurement of indicated gene expressions. (n=3) (B) The expressions of genes associated with Treg induction were evaluated among whole adipose tissues, mature adipocytes, SVF, macrophages, preadipocytes, hematopoietic cells and endothelial cells isolated from adipose tissues. (n=4) (C) Isolated primary adipose tissue macrophages were stimulated with 100μM EPA or 5-HEPE for 24 hours followed by measurement of indicated gene expressions. (n=4) Data are represented as mean ±SEM.

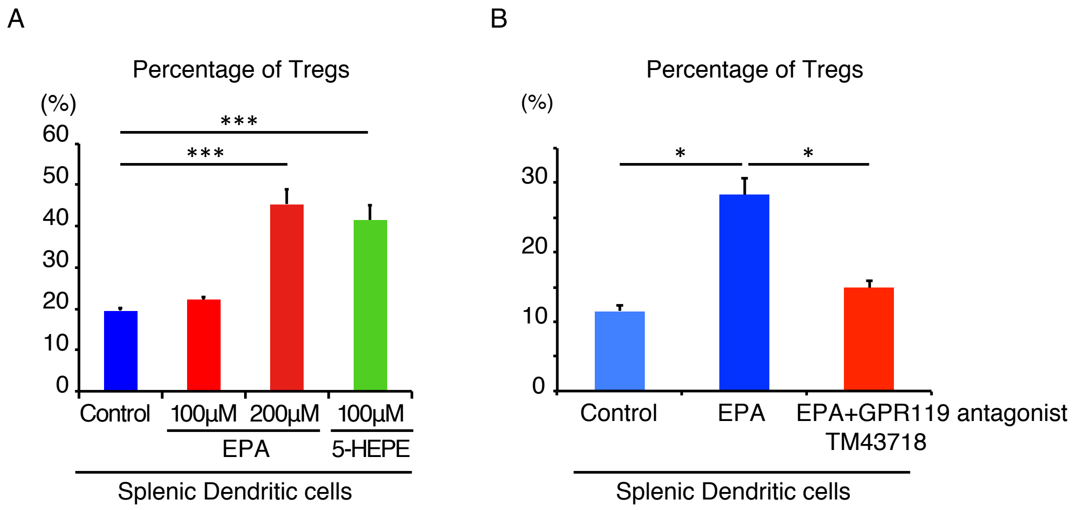

Supplementary figure 4 EPA and 5-HEPE augment Treg induction by splenic dendritic cells via GPR119.

(A) The effect of EPA and 5-HEPE on *in vitro* Treg induction by splenic dendritic cells. (n=3) (B) The effect of GPR119 antagonist TM43718 (10μM) on Treg induction by splenic dendritic cells. (n=3) Data are represented as mean ±SEM.

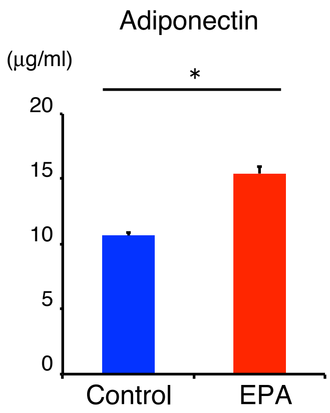

Supplementary figure 5 Serum adiponectin level was elevated in EPA-fed ob/ob mice.

5%EPA containing diet was fed to ob/ob mice for 4-5 weeks followed by measurement of serum adiponectin. Serum adiponectin levels of EPA fed ob/ob mice fasted for 4 hours. (n=8) Data are represented as mean ±SEM.
